# 5-(2,3,4,5,6-Penta­fluoro­phen­yl)-1,3,4-thia­diazol-2-amine

**DOI:** 10.1107/S1600536810039723

**Published:** 2010-10-09

**Authors:** Peng Wang, Rong Wan, Jianqiang Zhang, Peng Yu, Qiu He

**Affiliations:** a Department of Applied Chemistry, College of Science, Nanjing University of Technology, No. 5 Xinmofan Road, Nanjing, Nanjing 210009, People’s Republic of China

## Abstract

The title compound, C_8_H_2_F_5_N_3_S, was synthesized by the reaction of perfluoro­benzoic acid and thio­semicarbazide. The dihedral angle between the thia­diazole and perfluoro­phenyl ring is 35.41 (6)°. In the crystal, inter­molecular N—H⋯N hydrogen bonds link the mol­ecules, forming a three-dimensional network.

## Related literature

For the fungicidal and herbicidal activity of thia­diazole deriv­atives, see: Chen *et al.* (2000 ▶); Kidwai *et al.* (2000 ▶); Vicentini *et al.* (1998 ▶) and for their insecticidal activity, see: Arun *et al.* (1999 ▶); Wasfy *et al.* (1996 ▶). For bond-length data, see: Allen *et al.* (1987 ▶).
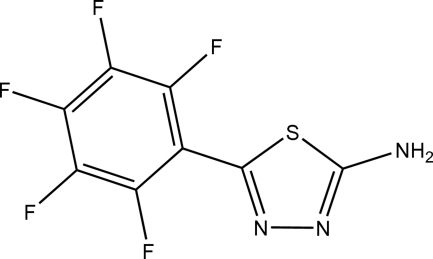


## Experimental

### 

#### Crystal data

C_8_H_2_F_5_N_3_S
*M* *_r_* = 267.19
Monoclinic, 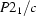

*a* = 11.897 (2) Å
*b* = 7.0680 (14) Å
*c* = 11.553 (2) Å
β = 104.66 (3)°
*V* = 939.8 (3) Å^3^
*Z* = 4
Mo *K*α radiation
μ = 0.40 mm^−1^
*T* = 293 K
0.30 × 0.20 × 0.10 mm

#### Data collection

Enraf–Nonius CAD-4 diffractometer
Absorption correction: ψ scan (North *et al.*, 1968 ▶) *T*_min_ = 0.889, *T*_max_ = 0.961
3428 measured reflections
1709 independent reflections
1283 reflections with *I* > 2σ(*I*)
*R*_int_ = 0.064
3 standard reflections every 200 reflections  intensity decay: 1%

#### Refinement

*R*[*F* ^2^ > 2σ(*F* ^2^)] = 0.043
*wR*(*F* ^2^) = 0.128
*S* = 1.00
1709 reflections
154 parameters
H-atom parameters constrained
Δρ_max_ = 0.27 e Å^−3^
Δρ_min_ = −0.29 e Å^−3^

### 

Data collection: *CAD-4 EXPRESS* (Enraf–Nonius, 1994 ▶); cell refinement: *CAD-4 EXPRESS*; data reduction: *XCAD4* (Harms & Wocadlo, 1995 ▶); program(s) used to solve structure: *SHELXS97* (Sheldrick, 2008 ▶); program(s) used to refine structure: *SHELXL97* (Sheldrick, 2008 ▶); molecular graphics: *SHELXTL* (Sheldrick, 2008 ▶); software used to prepare material for publication: *SHELXL97*.

## Supplementary Material

Crystal structure: contains datablocks global, I. DOI: 10.1107/S1600536810039723/er2081sup1.cif

Structure factors: contains datablocks I. DOI: 10.1107/S1600536810039723/er2081Isup2.hkl

Additional supplementary materials:  crystallographic information; 3D view; checkCIF report

## Figures and Tables

**Table 1:** Hydrogen-bond geometry (Å, °)

*D*—H⋯*A*	*D*—H	H⋯*A*	*D*⋯*A*	*D*—H⋯*A*
N1—H1*A*⋯N2^i^	0.86	2.18	3.001 (4)	160
N1—H1*B*⋯N3^ii^	0.86	2.19	3.013 (3)	161

Symmetry codes: (i) 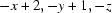; (ii) 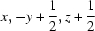.

**Fig. 1.**
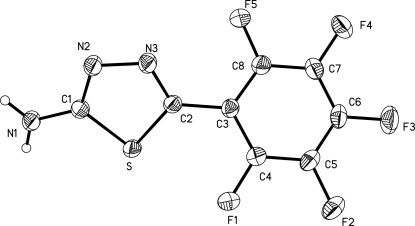
A view of the molecular structure of (I). Displacement ellipsoids are drawn at the 50% probability level.

**Fig. 2.**
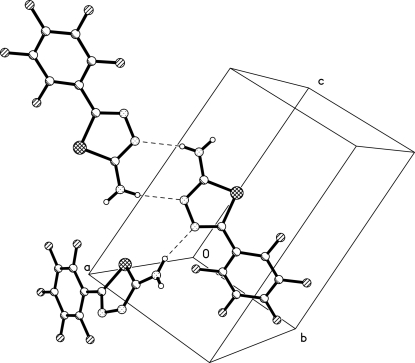
Partial packing view showing the hydrogen-bonded network. Dashed lines indicate intermolecular N—H···N hydrogen bonds.

## supplementary crystallographic information

### Comment

Thiadiazole derivatives containing the thiazolidinone unit are of great interest because of their chemical and pharmaceutical properties. Some derivatives have fungicidal and herbicidal activities (Chen *et al.*, 2000; Kidwai *et al.*, 2000; Vicentini *et al.*, 1998); some show insecticidal activities (Arun *et al.*, 1999; Wasfy *et al.*, 1996).

We report here the crystal structure of the title compound,(I). The molecular structure of (I) is shown in Fig.1, in which the bond lengths (Allen *et al.*, 1987) and angles are generally within normal ranges. Ring(C1/S/C2/N3/N2) is planar, and the mean deviation from plane is 0.0012 Angstroms. The dihedral angle between the thiadiazole and perfluorophenyl ring is 35.41 (6)°. In the crystal structure, intermolecular N—H···N hydrogen bonds (Table 1) link the molecules to form a three-dimensional network (Fig. 2), in which they may be effective in the stabilization of the structure.

### Experimental

Perfluorobenzoic acid (5 mmol) and thiosemicarbazide (5 mmol) were added in toluene (50 ml), which is heated under reflux for 4 h. The reaction mixture was left to cool to room temperature, poured into ice water, filtered, and the filter cake was crystallized from acetone to give pure compound (I) (m.p. 523–525 K). Crystals of (I) suitable for X-ray diffraction were obtained by slow evaporation of an acetone solution.

### Refinement

All H atoms bonded to the C atoms were placed geometrically at the distances of 0.93–0.97 Å and included in the refinement in riding motion approximation with *U*_iso_(H) = 1.2 or 1.5*U*_eq_ of the carrier atom.

### Crystal data

**Table:**

C_8_H_2_F_5_N_3_S	*F*(000) = 528
*M**_r_* = 267.19	*D*_x_ = 1.888 Mg m^−^^3^
Monoclinic, *P*2_1_/*c*	Melting point = 523–525 K
Hall symbol: -P 2ybc	Mo *K*α radiation, λ = 0.71073 Å
*a* = 11.897 (2) Å	Cell parameters from 25 reflections
*b* = 7.0680 (14) Å	θ = 9–13°
*c* = 11.553 (2) Å	µ = 0.40 mm^−^^1^
β = 104.66 (3)°	*T* = 293 K
*V* = 939.8 (3) Å^3^	Block, colorless
*Z* = 4	0.30 × 0.20 × 0.10 mm

### Data collection

**Table:**

Enraf–Nonius CAD-4 diffractometer	1283 reflections with *I* > 2σ(*I*)
Radiation source: fine-focus sealed tube	*R*_int_ = 0.064
graphite	θ_max_ = 25.3°, θ_min_ = 1.8°
ω/2θ scans	*h* = −14→0
Absorption correction: ψ scan (North *et al.*, 1968)	*k* = −8→8
*T*_min_ = 0.889, *T*_max_ = 0.961	*l* = −13→13
3428 measured reflections	3 standard reflections every 200 reflections
1709 independent reflections	intensity decay: 1%

### Refinement

**Table:**

Refinement on *F*^2^	Primary atom site location: structure-invariant direct methods
Least-squares matrix: full	Secondary atom site location: difference Fourier map
*R*[*F*^2^ > 2σ(*F*^2^)] = 0.043	Hydrogen site location: inferred from neighbouring sites
*wR*(*F*^2^) = 0.128	H-atom parameters constrained
*S* = 1.00	*w* = 1/[σ^2^(*F*_o_^2^) + (0.080*P*)^2^] where *P* = (*F*_o_^2^ + 2*F*_c_^2^)/3
1709 reflections	(Δ/σ)_max_ < 0.001
154 parameters	Δρ_max_ = 0.27 e Å^−^^3^
0 restraints	Δρ_min_ = −0.29 e Å^−^^3^

### Special details

**Table:**

Geometry. All e.s.d.'s (except the e.s.d. in the dihedral angle between two l.s. planes) are estimated using the full covariance matrix. The cell e.s.d.'s are taken into account individually in the estimation of e.s.d.'s in distances, angles and torsion angles; correlations between e.s.d.'s in cell parameters are only used when they are defined by crystal symmetry. An approximate (isotropic) treatment of cell e.s.d.'s is used for estimating e.s.d.'s involving l.s. planes.
Refinement. Refinement of *F*^2^ against ALL reflections. The weighted *R*-factor *wR* and goodness of fit *S* are based on *F*^2^, conventional *R*-factors *R* are based on *F*, with *F* set to zero for negative *F*^2^. The threshold expression of *F*^2^ > σ(*F*^2^) is used only for calculating *R*-factors(gt) *etc*. and is not relevant to the choice of reflections for refinement. *R*-factors based on *F*^2^ are statistically about twice as large as those based on *F*, and *R*- factors based on ALL data will be even larger.

### Fractional atomic coordinates and isotropic or equivalent isotropic displacement parameters (Å^2^)

**Table:**

	*x*	*y*	*z*	*U*_iso_*/*U*_eq_	
S	0.82940 (7)	0.06662 (10)	0.05893 (6)	0.0461 (3)	
F1	0.78775 (15)	−0.3360 (2)	−0.00245 (14)	0.0569 (5)	
C1	0.9155 (2)	0.2602 (4)	0.0479 (2)	0.0404 (6)	
N1	0.9659 (2)	0.3683 (4)	0.1416 (2)	0.0524 (6)	
H1A	1.0105	0.4601	0.1330	0.063*	
H1B	0.9537	0.3459	0.2106	0.063*	
C2	0.8165 (2)	0.0294 (4)	−0.0925 (2)	0.0389 (6)	
F2	0.65099 (17)	−0.6042 (2)	−0.12921 (18)	0.0685 (6)	
N2	0.9298 (2)	0.2855 (4)	−0.06011 (19)	0.0472 (6)	
F3	0.53266 (17)	−0.5342 (3)	−0.35867 (18)	0.0747 (6)	
N3	0.8725 (2)	0.1519 (3)	−0.13946 (19)	0.0451 (6)	
C3	0.7437 (2)	−0.1198 (4)	−0.1630 (2)	0.0387 (6)	
F4	0.55308 (16)	−0.1942 (3)	−0.45890 (14)	0.0689 (6)	
C4	0.7312 (2)	−0.2972 (4)	−0.1154 (2)	0.0429 (6)	
F5	0.68393 (17)	0.0780 (2)	−0.33282 (15)	0.0609 (5)	
C5	0.6609 (3)	−0.4358 (4)	−0.1800 (3)	0.0489 (7)	
C6	0.6009 (2)	−0.4016 (4)	−0.2954 (3)	0.0502 (7)	
C7	0.6116 (2)	−0.2282 (4)	−0.3459 (2)	0.0482 (7)	
C8	0.6812 (2)	−0.0900 (4)	−0.2800 (2)	0.0453 (7)	

### Atomic displacement parameters (Å^2^)

**Table:**

	*U*^11^	*U*^22^	*U*^33^	*U*^12^	*U*^13^	*U*^23^
S	0.0633 (5)	0.0429 (4)	0.0347 (4)	−0.0129 (3)	0.0171 (3)	−0.0005 (3)
F1	0.0665 (10)	0.0469 (10)	0.0521 (10)	−0.0049 (8)	0.0052 (8)	0.0130 (8)
C1	0.0472 (14)	0.0406 (15)	0.0352 (13)	−0.0044 (12)	0.0139 (12)	−0.0027 (11)
N1	0.0725 (16)	0.0530 (15)	0.0339 (12)	−0.0221 (13)	0.0176 (11)	−0.0081 (10)
C2	0.0477 (14)	0.0358 (14)	0.0354 (13)	−0.0023 (11)	0.0147 (12)	−0.0012 (11)
F2	0.0801 (13)	0.0418 (10)	0.0824 (14)	−0.0148 (9)	0.0184 (11)	0.0058 (9)
N2	0.0600 (14)	0.0494 (14)	0.0368 (12)	−0.0185 (11)	0.0206 (11)	−0.0092 (10)
F3	0.0815 (13)	0.0659 (13)	0.0740 (13)	−0.0330 (10)	0.0145 (11)	−0.0233 (10)
N3	0.0586 (14)	0.0442 (13)	0.0362 (12)	−0.0136 (11)	0.0191 (11)	−0.0087 (10)
C3	0.0450 (14)	0.0351 (14)	0.0378 (13)	−0.0027 (11)	0.0139 (11)	−0.0040 (11)
F4	0.0762 (12)	0.0821 (14)	0.0411 (9)	−0.0162 (11)	0.0012 (8)	−0.0056 (9)
C4	0.0458 (15)	0.0405 (15)	0.0426 (15)	−0.0010 (12)	0.0114 (12)	0.0025 (12)
F5	0.0840 (12)	0.0443 (10)	0.0477 (10)	−0.0082 (9)	0.0041 (9)	0.0086 (7)
C5	0.0553 (16)	0.0349 (15)	0.0603 (18)	−0.0060 (13)	0.0217 (14)	−0.0005 (13)
C6	0.0508 (16)	0.0486 (17)	0.0531 (17)	−0.0159 (14)	0.0165 (14)	−0.0172 (13)
C7	0.0492 (16)	0.0547 (18)	0.0406 (15)	−0.0051 (13)	0.0108 (13)	−0.0087 (13)
C8	0.0542 (16)	0.0404 (15)	0.0429 (15)	−0.0015 (12)	0.0155 (13)	−0.0008 (11)

### Geometric parameters (Å, °)

**Table:**

S—C1	1.733 (3)	N2—N3	1.372 (3)
S—C2	1.737 (3)	F3—C6	1.331 (3)
F1—C4	1.336 (3)	C3—C8	1.383 (4)
C1—N2	1.313 (3)	C3—C4	1.392 (4)
C1—N1	1.337 (3)	F4—C7	1.336 (3)
N1—H1A	0.8600	C4—C5	1.379 (4)
N1—H1B	0.8600	F5—C8	1.339 (3)
C2—N3	1.292 (3)	C5—C6	1.365 (5)
C2—C3	1.473 (4)	C6—C7	1.377 (4)
F2—C5	1.345 (3)	C7—C8	1.379 (4)
			
C1—S—C2	87.05 (12)	F1—C4—C5	118.0 (3)
N2—C1—N1	123.5 (2)	F1—C4—C3	119.5 (2)
N2—C1—S	113.5 (2)	C5—C4—C3	122.4 (3)
N1—C1—S	122.92 (19)	F2—C5—C6	120.1 (3)
C1—N1—H1A	120.0	F2—C5—C4	120.0 (3)
C1—N1—H1B	120.0	C6—C5—C4	119.9 (3)
H1A—N1—H1B	120.0	F3—C6—C5	120.5 (3)
N3—C2—C3	122.8 (2)	F3—C6—C7	120.2 (3)
N3—C2—S	113.4 (2)	C5—C6—C7	119.3 (2)
C3—C2—S	123.72 (19)	F4—C7—C6	119.5 (2)
C1—N2—N3	112.3 (2)	F4—C7—C8	120.3 (3)
C2—N3—N2	113.8 (2)	C6—C7—C8	120.2 (3)
C8—C3—C4	116.0 (2)	F5—C8—C7	117.2 (2)
C8—C3—C2	121.8 (2)	F5—C8—C3	120.8 (2)
C4—C3—C2	122.2 (2)	C7—C8—C3	122.1 (3)
			
C2—S—C1—N2	−0.2 (2)	F1—C4—C5—C6	179.5 (3)
C2—S—C1—N1	−177.7 (3)	C3—C4—C5—C6	0.3 (4)
C1—S—C2—N3	0.2 (2)	F2—C5—C6—F3	−0.4 (4)
C1—S—C2—C3	−176.3 (2)	C4—C5—C6—F3	−179.9 (3)
N1—C1—N2—N3	177.7 (3)	F2—C5—C6—C7	179.7 (2)
S—C1—N2—N3	0.2 (3)	C4—C5—C6—C7	0.2 (4)
C3—C2—N3—N2	176.4 (2)	F3—C6—C7—F4	0.0 (4)
S—C2—N3—N2	−0.2 (3)	C5—C6—C7—F4	179.9 (3)
C1—N2—N3—C2	0.0 (4)	F3—C6—C7—C8	179.3 (3)
N3—C2—C3—C8	−34.3 (4)	C5—C6—C7—C8	−0.8 (4)
S—C2—C3—C8	141.9 (2)	F4—C7—C8—F5	2.1 (4)
N3—C2—C3—C4	147.3 (3)	C6—C7—C8—F5	−177.2 (3)
S—C2—C3—C4	−36.4 (4)	F4—C7—C8—C3	−179.6 (2)
C8—C3—C4—F1	−179.2 (2)	C6—C7—C8—C3	1.1 (4)
C2—C3—C4—F1	−0.8 (4)	C4—C3—C8—F5	177.6 (2)
C8—C3—C4—C5	0.0 (4)	C2—C3—C8—F5	−0.8 (4)
C2—C3—C4—C5	178.4 (3)	C4—C3—C8—C7	−0.6 (4)
F1—C4—C5—F2	−0.1 (4)	C2—C3—C8—C7	−179.1 (3)
C3—C4—C5—F2	−179.3 (2)		

### Hydrogen-bond geometry (Å, °)

**Table:**

*D*—H···*A*	*D*—H	H···*A*	*D*···*A*	*D*—H···*A*
N1—H1A···N2^i^	0.86	2.18	3.001 (4)	160
N1—H1B···N3^ii^	0.86	2.19	3.013 (3)	161

Symmetry codes: (i) −*x*+2, −*y*+1, −*z*; (ii) *x*, −*y*+1/2, *z*+1/2.
